# Impact of periprosthetic femoral fractures on frailty, mobility and outcomes in hip arthroplasty

**DOI:** 10.1186/s13018-025-06446-z

**Published:** 2025-11-07

**Authors:** Roy Stanley, R. Roodt, M. Umar, D. Moloney, S. McCarthy, C. Hehir, U. Kelleher, R. Doyle, A. Molloy, C. Hurson

**Affiliations:** 1 Department of Trauma & Orthopaedic Surgery, St Vincent’s University Hospital, Elm Park, Dublin 4, Dublin, Ireland; 2 UCD School of Medicine & Medical Sciences, University College, Dublin, Ireland

**Keywords:** Periprosthetic fracture, Hip arthroplasty, Frailty, Mobility, Mortality

## Abstract

**Aim:**

The demand for total hip arthroplasty and hemiarthroplasty is rising, increasing the incidence of periprosthetic femoral fractures. This study aimed to assess clinical outcomes, including mortality, length of stay, and the impact of periprosthetic femoral fractures on mobility and frailty at one-year follow-up.

**Methods:**

A retrospective analysis of prospectively collected data was conducted looking at periprosthetic femoral fractures at a tertiary referral center from 2018 to 2024. The data collected included comorbidities, fracture classification, treatment method, length of stay, and discharge destination. The mortality rates at 30 days and one year were calculated. Mobility and frailty were assessed via the New Mobility Score and Clinical Frailty Scale before fracture and at one year. Statistical analysis included chi-square and Wilcoxon signed rank tests.

**Results:**

A total of *n* = 79 patients met the inclusion criteria (mean age 79.6 ± 9.5 years). There was a preponderance of females (35:44, M: F, *p* = 0.311). Vancouver B2 was the most common fracture pattern (*n* = 38). Surgical fixation was performed in *n* = 58 patients. Mortality rate at 30-day and one-year were 7.5% (*n* = 6) and 16.4% (*n* = 12) respectively. The mean Charlson Comorbidity Index was 4.39, with a score greater than 5 associated with higher one-year mortality (*p* = 0.031). Nursing home residency increased by 16%. The median New Mobility Score decreased from 7 to 5 (*p* < 0.001). The median Clinical Frailty Scale score increased from 4 to 5 (*p* < 0.001).

**Conclusion:**

Periprosthetic femoral fractures affect elderly, comorbid patients and are associated with high mortality. We observed measurable and significant decreases in mobility and frailty. Prompt treatment and early mobilization should be prioritized to improve outcomes.

## Introduction

The incidence of periprosthetic femoral fractures (PFFs) following total hip arthroplasty (THA) and hemiarthroplasty is increasing, presenting a significant challenge for orthopaedic care [1–3]. This trend is mirrored by the increasing number of primary procedures being performed [2]. The main risk factors include advanced age, female sex, and comorbidities such as osteoporosis and rheumatoid arthritis [4]. Substantial morbidity, mortality, prolonged hospitalization and adverse events such as blood loss are associated with PFFs [5, 6]. Venous thromboembolism is a major complication after THA and PFFs. Low-dose aspirin or appropriately timed enoxaparin provides effective thromboprophylaxis with minimal bleeding and wound complications [7, 8]. There is a socioeconomic burden through increased demand for rehabilitation and long-term care, along with extended inpatient stays [9]. The advanced age and multimorbidities of patients presenting with PFFs complicate their management and increase mortality risk [10, 11]. The reported 30-day and one-year mortality rates range from 3.3 to 9.3% and 13.4–22.3%, respectively [12, 13]. The severity of fracture classification is linked with higher mortality rates [14]. The most recent Irish Hip Fracture database recorded 3983 hip fractures in 2023, 3% of which were PFFs [2].

A significant loss of mobility occurs following PFFs. Many patients fail to regain their prefracture walking status and rely on walking aids and prolonged rehabilitation [13]. The New Mobility Score (NMS) is a widely used and validated tool for assessing mobility. The NMS has shown a decline of 1.5 three years after PFF [14]. While previous studies have examined recovery and overall health decline following PFFs, none have objectively quantified changes in mobility status after one year.

Frailty is common in this cohort and is defined as a decline in multiple body systems, leading to a reduction in cognitive and physical reserves [15]. The Clinical Frailty Scale (CFS) is a strong predictor of mortality rates in PFFs [16]. However, its change after fracture has not been quantified. In hip fracture patients, frailty is linked with poorer outcomes, increased mortality and longer hospital stays [17].

The evidence quantifying the effect of PFFs on mobility and frailty is limited, and no study to date has simultaneously assessed both frailty and mobility numerically after PFFs. The primary aim of this study was to assess the morbidity and functional outcomes of patients with PFFs by evaluating their frailty and mobility at the one-year follow-up in comparison to their prefracture status. The secondary aim was to determine the associated mortality rate in patients treated at our tertiary referral center.

## Methods

We performed a retrospective interrogation of a prospectively maintained database using data gathered at our tertiary referral center. Patients who sustained a PFF between January 1st 2018 and December 31st 2024, were identified. The data sources included the local Hip Fracture Database and local Orthogeriatric Database, with subsequent reviews of theatre lists and clinical notes undertaken.

The inclusion criterion was patients with a confirmed PFF around a THA or hemiarthroplasty that presented to our tertiary referral center. Patients were required to have sufficient records documenting follow-up data at one year or until death. The exclusion criteria included fractures around the knee arthroplasty site, nonarthroplasty implants (e.g., dynamic hip screws or intramedullary nails), incomplete records surrounding the CFS or NMS, lack of 1-year follow-up, and fractures around a trauma fixator.

Patient demographics, including age, sex, preadmission residence, and comorbidities were recorded. The Charlson comorbidity index (CCI) was calculated to evaluate comorbidities and their relationship with mortality [18]. Radiological images were reviewed by two senior orthopaedic consultants to determine the arthroplasty type (hemi- or total) and the type of fracture pattern via the Vancouver Classification System [19]. Both established whether patients were managed conservatively or surgically. Surgical interventions were categorized as open reduction internal fixation (ORIF), ORIF with stem revision, stem revision alone or proximal femur replacement. Stem revision combined with cerclage cables was classified as stem revision alone, as cerclage cables are considered supplementary fixation. All operations were performed by consultant orthopaedic surgeons via conventional techniques, without the assistance of artificial intelligence or robotic systems. Patients who were deemed medically unfit for any surgical intervention were classified as being managed conservatively. The length of inpatient stay was recorded in days.

Prefracture frailty was assessed via the nine-point CFS, a validated tool ranging from 1 (very fit) to 9 (terminally ill) on the basis of comorbidity, cognition, and functional status, with higher scores indicating greater frailty [20]. Prefracture mobility was measured via the NMS, which evaluates indoor walking, outdoor walking and shopping ability. Each item is scored 0–3, for a total score of 0 (immobile) to 9 (fully mobile) [21]. Discharge destinations were recorded as either home or nursing home/offsite rehabilitation facilities. At the one-year follow-up, patients’ current residence and any revision surgeries were recorded, and both the NMS and CFS were calculated. Both the CFS and NMS assessments were conducted by either an orthogeriatric consultant or a registrar. For patients who died within the year, the date of death was recorded enabling the calculation of 30-day and one-year mortality rates.

This study aimed to assess the clinical outcomes of patients with PFFs. The primary outcomes included assessments of frailty and mobility before fracture and at the one-year follow-up, with the aim of evaluating whether PFFs impacted these measures. The secondary outcome was the mortality rate. Statistical analyses were performed via Jamovi software. Categorical data were analyzed via the chi-square test. Pre- and postfracture frailty and mobility scores were compared via the Wilcoxon signed-rank test. Patients who died within one year were excluded from this analysis.

## Results

A total of 123 patients, *n* = 79 of whom met the inclusion criteria, sustained PFF between 2018 and 2024. The mean age was 79.6 years (± 9.5 years), ranging from 57 to 97 years. There was a preponderance of females, with *n* = 44 females and *n* = 35 males (*p* = 0.311). A total of 83.5% (*n* = 66) were admitted from home, with *n* = 12 transferred from a nursing home, and one sustained a fracture while already in the hospital. Patients with a THA accounted for *n* = 61 patients, and *n =* 18 patients had a hemiarthroplasty. Fractures were classified as A = 17, B1 = 8, B2 = 38, B3 = 8, C = 8 (Table 1). Surgical fixation was performed in *n* = 58 patients, including *n* = 43 patients who underwent ORIF, seven patients who underwent stem revision plus ORIF and seven underwent stem revision alone (Table 1). Nonoperative management was used in 21 patients either due to fracture configuration or who were deemed unfit medially for surgery, with 71.4% being from fracture classification A (*n* = 15), two in the B1 group and 4 in the B2 group. The median length of inpatient hospital stay was 14 days (range 1–136 days).

**Table 1:** Management of different patterns of fracture according to the Vancouver classification system [19]

Fracture type	Management
Type A (*n* = 17)	Non-operative = 15 ORIF = 2
Type B1 (*n* = 8)	Non-operative = 2 ORIF = 6
Type B2 (*n* = 38)	Non-operative = 4 ORIF = 25 Stem revision = 4 Stem revision + ORIF = 5
Type B3 (*n* = 8)	ORIF = 2 Stem revision = 3 Stem revision + ORIF = 2 Proximal Femur Replacement = 1
Type C (*n* = 8)	ORIF = 8

**Fig. 1:**
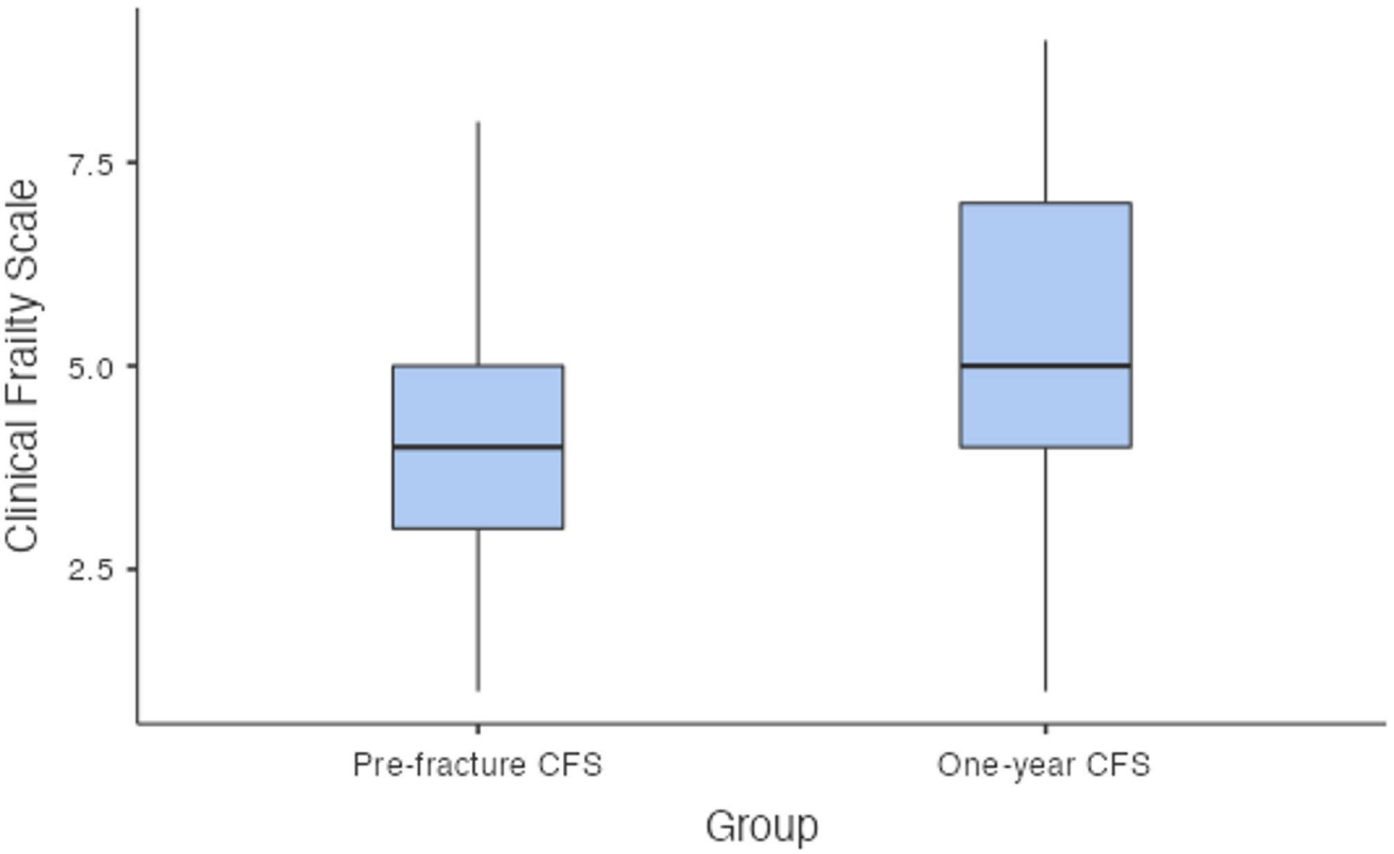
Box plot illustrating the change in the clinical frailty scale score from prefracture to one year post-fracture (*p* < 0.001)

**Fig. 2:**
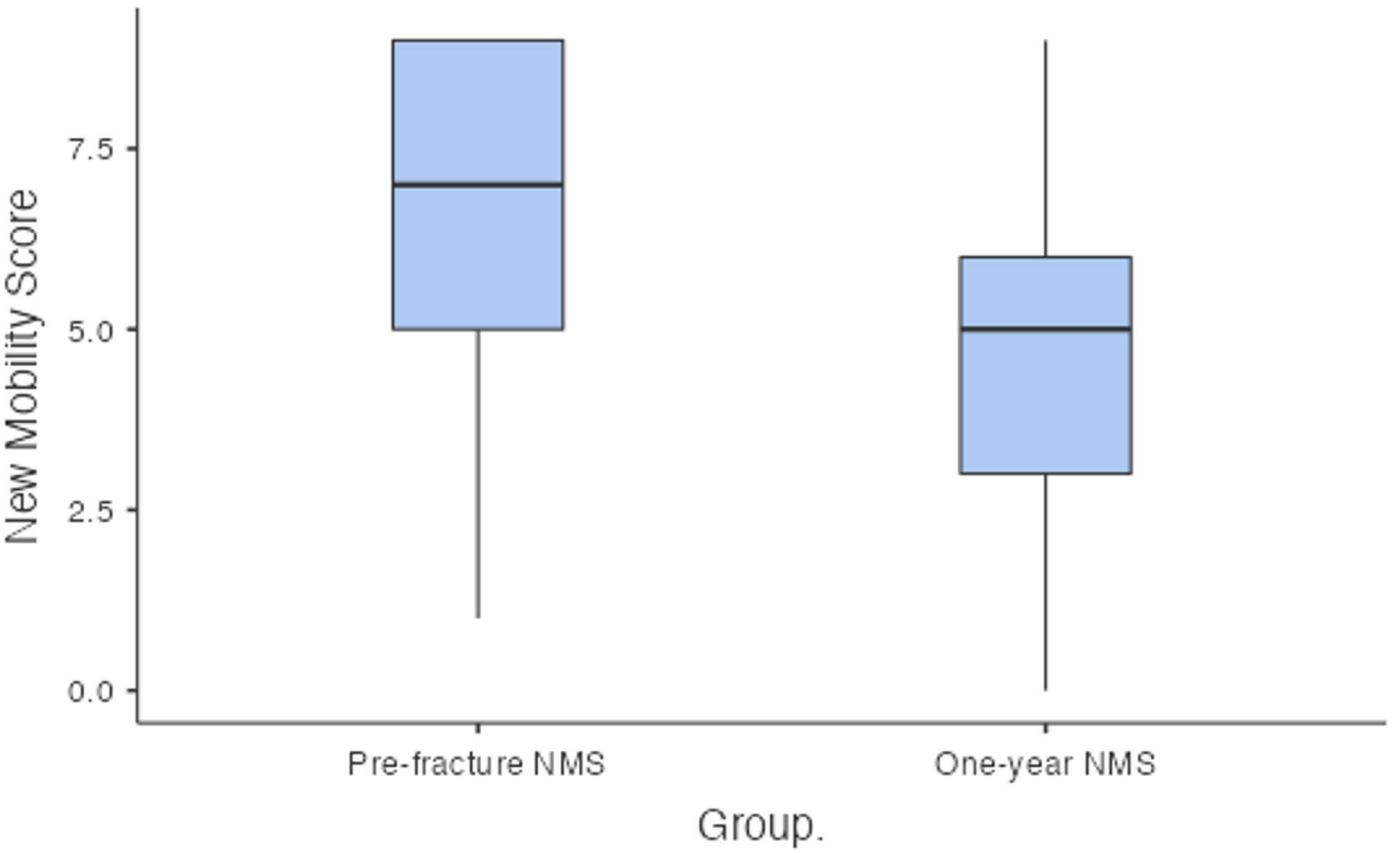
Box plot illustrating the change in the new mobility score from prefracture to one-year post-fracture (*p* < 0.001)

The 30-day mortality rate was 7.5% (*n* = 6). The one-year mortality rate was 16.4% (*n* = 12). The mean CCI was 4.39; a CCI of greater than or equal to 5 was associated with a higher one-year mortality rate (*p* = 0.031). In terms of discharge destination, 12 patients returned home, 59 were discharged directly to a nursing home or off-site rehabilitation facility, and eight died while in the hospital. The median prefracture CFS was 4 (IQR: 3–5) (Fig. 1). The median prefracture NMS was 7 (IQR: 5–9) (Fig. 2).

A total of *n =* 61 patients were included in the one-year follow-up. At this time, 67.2% (*n =* 41) were living at home and 20 were living in a nursing home, representing a 16% increase in nursing home residency compared with prefracture status. No patients required revision surgery. The median one-year CFS was 5 (IQR: 4–7) (Fig. 1). There was a significant decline in frailty over one year. The mean difference between the prefracture and one-year CFS values was − 1.50 (95% CI [− 2, − 1.5], *p* < 0.001). There was also a significant reduction in mobility over one year. The median one-year NMS was 5 (IQR: 3–6) (Fig. 2). The mean difference between prefracture NMS and one-year NMS was 2.5 (95% CI [2, 3], *p* < 0.001).

## Discussion

PFFs represent a challenging complication following THA and hemiarthroplasty and are expected to continue contributing substantially to the orthopaedic workload, as joint arthroplasty has become increasingly common. This trend is driven by an aging population, with a projected 150% increase in people over 65 years of age over the next 30 years [22].

In our study, the 30-day mortality rate was 7.5%, which is consistent with reports in the literature ranging from 3.3 to 9.3%. The one-year mortality rate was 16.4%, which is also in line with published data (13.4–22.3%) [12–14]. Our study only assessed 30-day and one-year mortality rates, but previous research has reported a three-year mortality rate of 48%, with higher mortality rates with increasing fracture severity [14]. The COMPOSE study reported 30-day and one-year mortality rates of 5.2% and 21.0%, respectively [23]. Relative survival following PFF has been shown to be worse than that after other indications for revision THA, including aseptic loosening, infection or dislocation [24]. The elevated mortality rate in our cohort is not surprising, given the advanced mean age of nearly 80 years and a mean CCI of 4.39. Patients with a CCI greater than or equal to 5 had significantly higher one-year mortality rates, supporting previous evidence linking a greater comorbidity burden with poorer outcomes [18, 25]. Advanced age is a well-established risk factor for PFFs [4, 26]. In contrast to previous findings within the literature, sex was not associated with increased PFF risk in our cohort, which aligns with the findings of only one prior study [26].

Discharge destination data highlight the long-term impact of PFFs on independence. A total of 59 patients were discharged to nursing homes or offsite rehabilitation facilities, whereas 12 patients were admitted initially from nursing homes. The COMPOSE study reported that 61% of patients admitted initially from home were discharged back to their own residence [23]. At the one-year follow-up, 20 patients were residing in nursing homes—representing a 16% increase and highlighting the profound effect on patient independence. While institutionalization has not been widely studied in PFFs, hip fractures alone are associated with rates of 10–20% [27].

Our median length of hospital stay was 14 days, which is comparable to the 15 days reported in the COMPOSE study [23]. Length of in-hospital stay is a major contributor to healthcare costs in these PFFs, accounting for up to 80% of total costs in one study, which reported a mean length of stay of 36 days [1]. In a frail elderly cohort, prolonged hospital stays are often unavoidable due to the recovery process, unlike elective or revision THA, where discharge planning can typically be arranged prior to admission.

With respect to surgical management, more than 75% of patients underwent fixation alone rather than stem revision. Fixation-only strategies are associated with reduced blood loss, shorter operative times, and decreased lengths of stay because of their less invasive nature [28]. Almost half of the fractures were classified as Vancouver B2. While the COMPOSE study reported 55% B2 fractures, 77% of these fractures underwent revision, whereas only 24% did in our center [23]. The reasons for choosing ORIF over revision was not recorded at the time of surgery, which limits our ability to determine whether surgical decisions followed standard guidelines. One patient required proximal femur replacement because of compromised bone quality, precluding reliable fixation or revision. Fracture pattern type A fractures were predominantly managed conservatively (88%), which is consistent with standard practice, unless the trochanter fracture is displaced by more than 2 cm or the prosthesis is unstable [29]. None of the patients in our cohort required revision surgery, in contrast to the COMPOSE study which reported a one-year reoperation rate of 5.6% [23]. The low revision rate in our study may be due to the short follow-up, or careful preoperative planning of procedures. All operations were performed by consultant orthopaedic surgeons via conventional techniques, without the assistance of artificial intelligence or robotic systems. While robotic-assisted THA has generally demonstrated superior accuracy and safety compared with conventional approaches [30, 31], its application has not yet been studied in the management of PFFs.

Functionally, our cohort experienced significant declines in mobility and frailty at one year. The median NMS decreased from 7 to 5, reflecting reduced mobility. Previous studies have shown that it is difficult for patients to regain their prefracture walking ability, with a substantial portion relying on walking aids and approximately 58% failing to return to their prefracture walking status [13]. NMS has been shown to decrease by approximately 1.5 from the preoperative period to the last follow-up at 3 years [14]. Similarly, the median CFS increased from 4 to 5, indicating greater frailty at one year. Frailty has been linked with increased mortality [16], yet its change has not previously been quantified via a numerical scale. Self-sufficiency scores and functional scores such as the Harris hip score, indicate better outcomes for Vancouver A fractures than for B or C fractures [14]; however our study did not perform sub-group analyses. To our knowledge, this is the first study to quantify changes in both mobility and frailty numerically for PFF patients after one year, demonstrating that even with appropriate surgical management, patients rarely return to their prefracture functional status.

Our study is not without its limitations. As a retrospective study design, it is dependent on the quality and completeness of existing records. As it was conducted at a single center, the sample size was relatively small. Excluding patients from the functional outcome analysis who died within one year may have introduced survivorship bias, potentially leading to an overestimation of postfracture mobility and frailty by selectively analyzing a healthier subset of the cohort. The Vancouver classification was assessed by two expert orthopaedic consultants, but human error remains possible. Although the CFS and NMS are simple scoring tools, the lack of consistent investigators may have introduced inter-rater bias. Statistical analyses were unadjusted and did not account for covariates such as preoperative functional status or indication for primary arthroplasty; these factors likely influenced outcomes. Analyzing outcomes according to the Vancouver classification could provide a clearer understanding of how fracture type individually affects mobility and frailty. Despite these limitations, this is the first study to compare prefracture and one-year mobility and frailty in PFFs on a numerical scale. Future studies could compare the outcomes in PFFs with those of conventional hip fractures.

## Conclusions

Periprosthetic femoral fractures pose increasing challenges in the aging, comorbid population. Our study highlights the high mortality rate and significant declines in mobility and frailty experienced by this already high-risk population group. These findings emphasize the need for healthcare systems to allocate resources appropriately and prioritize treatment strategies to optimize long-term outcomes.

## Data Availability

The anonymised datasets analyzed during the current study are available from the corresponding author upon reasonable request.
